# Performances of rapid and connected salivary RT-LAMP diagnostic test for SARS-CoV-2 infection in ambulatory screening

**DOI:** 10.1038/s41598-022-04826-7

**Published:** 2022-02-18

**Authors:** Francisco Santos Schneider, Laurence Molina, Marie-Christine Picot, Nicolas L’Helgoualch, Julien Espeut, Pierre Champigneux, Mellis Alali, Julie Baptiste, Lise Cardeur, Christophe Carniel, Martin Davy, Daniel Dedisse, Benjamin Dubuc, Hugo Fenech, Vincent Foulongne, Carole Fruchart Gaillard, Florence Galtier, Alain Makinson, Grégory Marin, Raissa Medina Santos, David Morquin, Alimata Ouedraogo, Alexandra Prieux Lejeune, Marine Quenot, Pierre Keiflin, Francisco Checa Robles, Carolina Rodrigues Rego, Nicolas Salvetat, Charline Trento, Diana Vetter, Franck Molina, Jacques Reynes

**Affiliations:** 1 Sys2Diag UMR9005 CNRS ALCEN, Cap Gamma, Parc Euromédecine, 1682 rue de la Valsière, CS 40182, 34184 Montpellier, CEDEX 4, France; 2 SkillCell, Montpellier, France; 3 Clinical Research and Epidemiology Unit, Department of Medical Information, Montpellier University Hospital, University of Montpellier, Montpellier, France; 4 INSERM Centre Investigation Clinique 1411, University Hospital, Montpellier, France; 5 Vogo, Montpellier, France; 6 PCCEI, Univ Montpellier, INSERM, EFS, Univ Antilles, Montpellier, France; 7 CEA, INRAE, Department of Medicines and Healthcare Technologies (DMTS), University of Paris-Saclay, SIMoS, Gif-sur-Yvette, France; 8 Infectious Diseases Department, Montpellier University Hospital, Montpellier, France; 9 TransVIHMI, IRD, INSERM, University of Montpellier, Montpellier, France; 10 Department of Biochemistry and Immunology, Institute of Biological Sciences, Federal University of Minas Gerais, Belo Horizonte, Brazil

**Keywords:** Infectious-disease diagnostics, Diagnosis, Disease prevention, Epidemiology, Population screening

## Abstract

In the context of social events reopening and economic relaunch, sanitary surveillance of SARS-CoV-2 infection is still required. Here, we evaluated the diagnostic performances of a rapid, extraction-free and connected reverse-transcription loop-mediated isothermal amplification (RT-LAMP) assay on saliva. Nasopharyngeal (NP) swabs and saliva from 443 outpatients were collected simultaneously and tested by reverse-transcription quantitative PCR (RT-qPCR) as reference standard test. Seventy-one individuals (16.0%) were positive by NP and/or salivary RT-qPCR. Sensitivity and specificity of salivary RT-LAMP were 85.9% (95%CI 77.8–94.0%) and 99.5% (98.7–100%), respectively. Performances were similar for symptomatic and asymptomatic participants. Moreover, SARS-CoV-2 genetic variants were analyzed and no dominant mutation in RT-LAMP primer region was observed during the period of the study. We demonstrated that this RT-LAMP test on self-collected saliva is reliable for SARS-CoV-2 detection. This simple connected test with optional automatic results transfer to health authorities is unique and opens the way to secure professional and social events in actual context of economics restart.

## Introduction

The pandemic of coronavirus disease 2019 (COVID-19) calls for rapid, accurate and scalable diagnosis to circumvent the disease’s spread and safe reopen society. Reverse transcription quantitative-polymerase chain reaction (RT-qPCR) and antigen detection are the main employed diagnosis methods for the diagnosis of severe acute respiratory syndrome coronavirus 2 (SARS-CoV-2) infection, with RT-qPCR being the validated gold standard. This assay requires specialized and expensive instrumentation, trained personnel and supply-limited reagents. RT-qPCR requires RNA extraction, which is a bottleneck time-consuming step and normally returns results 24 h after sample collection.

RT-LAMP (reverse transcription loop-mediated isothermal amplification) is a rapid and portable technology requiring neither highly educated analyst nor specialized instruments (normally only a heat source is needed) rendering this technology an alternative to the RT-qPCR.

Nasopharyngeal (NP) and oropharyngeal (OP) swabs are the most commonly used samples for the diagnosis of SARS-CoV-2 infection. Sampling NP and OP swabs is invasive, painful and exposes healthcare-workers to contamination^1^. In contrast, saliva self-sampling is easy, non-invasive, and particularly suitable for children and elderly testing. Furthermore, saliva collection does not require specialized materials and exempts the use of personal protective equipment, saving time and costs. Saliva sampling being well accepted^2,3^, it is an adequate specimen for mass and home testing.

Likewise, SARS-CoV-2 has been detected at high loads in saliva of symptomatic and asymptomatic individuals and has also been shown to infect target cells^4–11^. Studies comparing saliva and NPS using nucleic acid amplification testing (NAAT) showed sensitivities varying from 69.2 to 100%^5,6,8,12,13^. A recent meta-analysis suggested that saliva and NPS NAAT diagnostic accuracies are similar^14^.

COVID-19 world sanitary situation remains heterogeneous mainly due to various anti-pandemic policies, resources and vaccination strategies^15^. For the next coming months the strategy for reopening and relaunching social and professional events will be central. In addition to middle/long term pandemic monitoring, testing population is still important since vaccine immunity and natural immunity are imperfect and social distancing is more flexibilized^16^. To this end, next to collective surveillance approaches (for instance sewage virus testing), authorities are looking for individual testing systems with higher population acceptability and efficiency. Hence, the ideal individual virus test specifications are: (1) Painless and simple sampling to avoid any reluctance from population for recurrent testing, (2) Easy, rapid and low cost to make it usable at very large scale and in any condition including without specialized laboratories and expertise, (3) Sensitive and specific to bring safety and (4) To be real time connected to allow immediate individual information as well as monitoring by local or national authorities^17^. This latest point is central to secure events or professional plants to return to activities. The development of a rapid salivary RT-LAMP that meets most of these points for SARS-CoV-2 detection would be a step forward in deploying a point-of-care test to safely re-open economies and prevent future outbreaks.

In this work, we evaluated the diagnostic performance of the rapid salivary RT-LAMP test on symptomatic and asymptomatic individuals in an ambulatory screening. Further, we also demonstrated that the salivary rapid RT-LAMP test is suitable for ambulatory testing and highly performant compared with gold standard.

## Results

### Description of the clinical reference test

The clinical reference standard used to define infected individuals was a composite reference test (CRTest), defined as a positive NP and/or a positive salivary RT-qPCR test. As previously described^18^, a RT-qPCR test was considered positive for SARS-CoV-2 when the cycle threshold (Ct) value was < 35 for at least one target. Negative individuals were mandatory to be negative for both specimens and constituted the reference negative group.

### Clinical characteristics of the cohort

**Figure 1:**
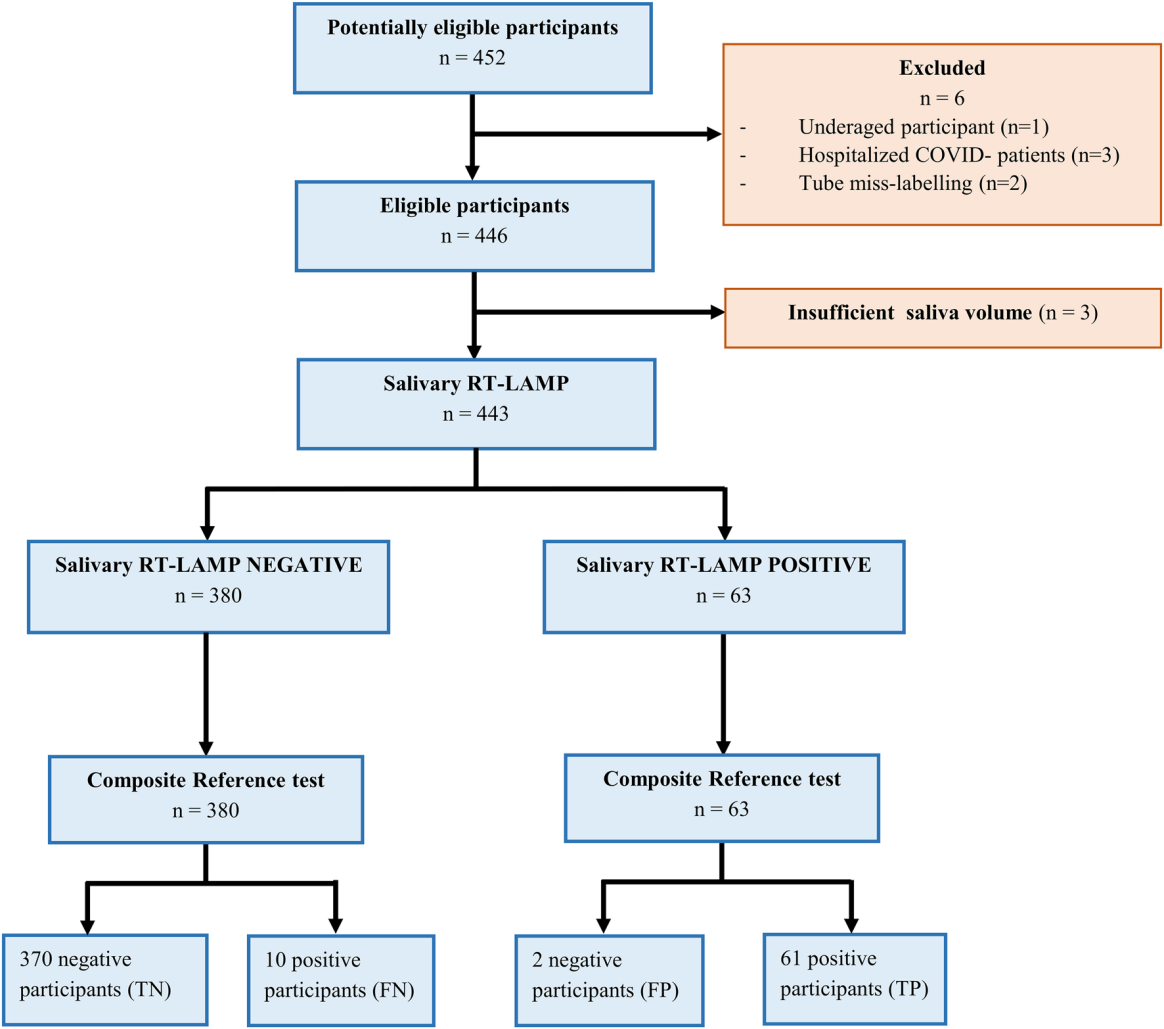
Study flow diagram. RT-LAMP, reverse transcription loop-mediated isothermal amplification; COVID, Coronavirus disease; TN, true negative; FN, false negative; TP, true positive; FP, false positive for salivary RT-LAMP.

A total of 452 individuals participated in the study and samples of 443 were analyzed (Fig. 1). Among the nine participants not included, three (0.67%) were not able to provide sufficient saliva volume. Demographics and clinical data are shown in Table 1. The female to male sex ratio was 1.46 and the mean age was 32.2 years (SD ± 14.0). At least one symptom was declared by 263 participants (59.4%) and 180 (40.6%) were totally asymptomatic. Most common symptoms were headache, asthenia, rhinorrhea, cough and myalgia (Table 1). Symptomatic participants presented only mild symptoms at inclusion. The frequency of symptoms was also analyzed on the negative and positive CRTest patients (Table 1). Six symptoms were significantly more frequent in positive individuals: olfactory and/or gustatory disorders, fever, myalgia, headache and vertigo. These symptoms are compatible with a SARS-CoV-2 infection.

**Table 1:** Demographic and clinical characteristics.

	Total population	Negative CRTest	Positive CRTest (active infection)	Test	*p-*value*
**Age, y, median (IQR)**	27.0 (21.0 ; 41.0)	27.0 (21.0; 40.0)	26.0 (21.0; 41.0)	WMW	0.8107
**Female sex**	263/443 (59.4)	224/372 (60.2)	39/71 (54.9)	CHI2	0.4060
**Symptoms on the day of sampling**	263/443 (59.4)	207/372 (55.6)	56/71 (78.9)	CHI2	0.0003
Fever	50/423 (11.8)	34/357 (9.5)	16/66 (24.2)	CHI2	0.0007
Cough	118/430 (27.4)	92/363 (25.3)	26/67 (38.8)	CHI2	0.0233
Expectoration/sputum	67/424 (15.8)	56/356 (15.7)	11/68 (16.2)	CHI2	0.9264
Thoracic pain	39/424 (9.2)	35/360 (9.7)	4/64 (6.2)	CHI2	0.3758
Dyspnea	73/426 (17.1)	59/358 (16.5)	14/68 (20.6)	CHI2	0.4099
Rhinorrhea	140/427 (32.8)	115/360 (31.9)	25/67 (37.3)	CHI2	0.3900
Myalgia	97/423 (22.9)	70/353 (19.8)	27/70 (38.6)	CHI2	0.0007
Weakness	143/426 (33.6)	114/359 (31.7)	29/67 (43.3)	CHI2	0.0666
Diarrhea	36/429 (8.4)	29/361 (8.0)	7/68 (10.3)	CHI2	0.5373
Nausea	37/424 (8.7)	27/356 (7.6)	10/68 (14.7)	CHI2	0.0566
Headache	155/433 (35.8)	120/363 (33.1)	35/70 (50.0)	CHI2	0.0068
Vertigo	51/429 (11.9)	37/362 (10.2)	14/67 (20.9)	CHI2	0.0131
Olfactory and/or Gustatory disorders	51/427 (11.9)	30/330 (8.3)	21/67 (31.3)	CHI2	< 0.0001
**Total number of symptoms on the day of sampling**					
None (asymptomatic)	180 (40.6)	165 (44.3)	15 (21.1)	CHI2	0.0011
1	45 (10.2)	39 (10.5)	6 (8.4)	CHI2	0.0011
2	38 (8.6)	30 (8.1)	8 (11.3)	CHI2	0.0011
3 or more	180 (40.6)	138 (37.1)	42 (59.1)	CHI2	0.0011

Unless otherwise indicated, data are reported as n/N (%). Denominators vary because of missing data for some participants.

*CRTest* composite reference test, *IQR* interquatile range.

*p-values for comparisons between patients with positive and negative PCR.

### Ergonomic of the salivary connected RT-LAMP-test

The RT-LAMP assay is a rapid, extraction-free salivary test and the colorimetric result can be read instantly by naked eye or automatically by EasyCOV® Reader software application.

EasyCOV Reader® can be used on a tablet or smartphone that are optionally connected to the Internet (via a WIFI, 4G or 5G connection) and the test result is stored on a Health Data Hosting (HDH) compliant database and can be transmitted to the sanitary authorities. If required, other data can be collected and stored simultaneously as patient identification number, date, time and geographical localization of the test.

### Diagnostic performance of rapid salivary RT LAMP assay

According to the CRTest, 71 individuals were positive (prevalence of 16.0%) (Table 2). RT-LAMP identified 61 (85.9%) of the positive samples. Ten positive individuals in CRTest were misdiagnosed by salivary RT-LAMP. Six of them presented salivary RT-qPCR Ct value ≥ 31 and negative NP RT-qPCR results while the other four were positive by NP RT-qPCR (Ct values between 23 and 33) and negative in salivary RT-qPCR. Among the 372 negative individuals in CRTest, 370 had a negative RT-LAMP. The sensitivity (Se) and the specificity (Sp) of RT-LAMP were 85.9% (95% CI 77.8–94.0) and 99.5% (95% CI 98.7–100), respectively (Table 3).

**Table 2:** Concordance table of salivary RT-LAMP results according to CRTest RT-qPCR results in total, symptomatic and asymptomatic populations.

	Salivary RT-LAMP								
	Total population			Symptomatic population			Asymptomatic popultation		
	Negative	Positive	Total	Negative	Positive	Total	Negative	Positive	Total
**CRTest RT-qPCR**									
Negative or C_t_ value ≥ 35	370	2	372	205	2	207	165	0	165
Positive (C_t_ value < 35)	10	61	71	9	47	56	1	14	15
Total	380	63	443	214	49	263	166	14	180

Data are reported as number of individuals.

*CRTest* composite reference test, *RT-qPCR* reverse transcription quantitative polymerase chain reaction, *RT-LAMP* reverse transcription loop-mediated isothermal amplification, *C*_*t*_ cycle threshold.

**Table 3:** Performances of RT-LAMP compared to different reference methods.

Reference method	Total (n)	Negative (n)	Positive n [%]	RT-LAMP				
				Sensitivity (%) (95%CI)	Specificity (%) (95%CI)	PPV (%) (95%CI)	NPV (%) (95%CI)	Accuracy (%) (95%CI)
**Composite reference test**								
Total population	443	372	71 [16.0%]	85.9 (77.8–94.0)	99.5 (98.7–100)	96.8 (92.5–100)	97.4 (95.8–99.0)	97.3 (95.8–98.8)
Symptomatic population	263	207	56 [21.3%]	83.9 (74.3–93.6)	99.0 (97.7–100)	95.9 (90.4–100)	95.8 (93.1–98.5)	95.8 (93.4–98.2)
Asymtomatic population	180	165	15 [8.3%]	93.3 (80.7–100)	100 (100–100)	100 (100–100)	99.4 (98.2–100)	99.4 (98.4–100
**NP RT-qPCR**	441	410	31 [7.0%]	87.1 (75.3–98.9)	91.5 (88.8–94.2)	43.5 (31.2–55.9)	98.9 (97.9–100.0)	91.0 (88.3–93.6)
**Salivary RT-qPCR**	443	376	67 [15.1%]	91.0 (84.2–97.9)	99.5 (98.7–100)	96.8 (92.5–100)	98.4 (97.2–99.7)	98.2 (96.9–99.4)

Data are reported as number of individuals or percentage, in parentheses are 95% CI and in brackets are prevalence.

*RT-qPCR* reverse transcription quantitative polymerase chain reaction, *RT-LAMP* reverse transcription loop-mediated isothermal amplification, *NP* nasopharyngeal, *CI* confidence interval, *PPV* Positive Predictive Value, *NPV* Negative Predictive Value.

### Performance of the salivary RT-LAMP in symptomatic and asymptomatic individuals

Among symptomatic participants (n = 263, 59.4%), RT-LAMP detected 47 of the 56 positive subjects (Se = 83.9%, 95% CI 74.3–93.6) (Tables 2 and 3). Two of the 207 negative symptomatic subjects were positive by salivary RT-LAMP (Sp = 99.0%, 95% CI 97.7–100) (Tables 2 and 3).

Concerning the asymptomatic participants (n = 180, 40.6%), 15 subjects (8.3%) were positive according to the CRTest. Only one subject was discordant, with a positive CRTest and a negative salivary RT-LAMP. All the other subjects had concordant results between EasyCOV® and the standard reference test. Performances were therefore Se = 93.3% (95% CI 80.7–100) and Sp = 100% (95% CI 100–100) (Tables 2 and 3).

### Salivary RT-LAMP sensitivity according to virus load

**Figure 2:**
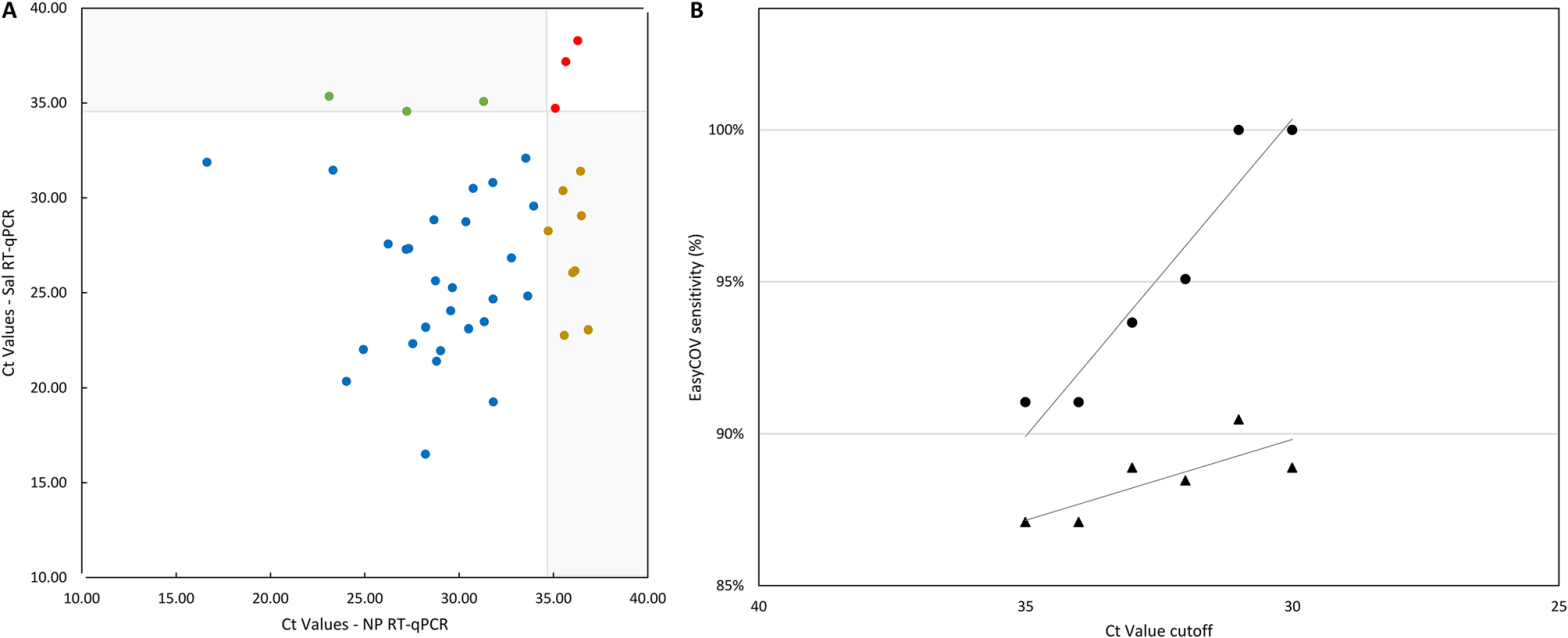
(A) Sensitivity of salivary RT-LAMP evaluated against NP and salivary RT-qPCR Ct values. Ct value cutoffs indicate the infectious status of individuals considered as positive/negative**.** Dots  show sensitivity (%) of RT-LAMP against salivary RT-qPCR (R^2^ = 0.92). Triangles  show sensitivity (%) of salivary RT-LAMP compared to NP RT-qPCR (R^2^ = 0.61). RT-qPCR, reverse transcription quantitative polymerase chain reaction; RT-LAMP, reverse transcription loop-mediated isothermal amplification; NP, nasopharyngeal; Sal, salivary; Ct, cycle threshold. (B) Comparison of Ct Value between NP and salivary RT-qPCR. Dots represent experimentally measured Ct values of nasopharyngeal (X) and salivary (Y) RT-qPCR. Positive concordant individuals (positive for both NP and salivary RT-qPCR) are in down left white corner . Negative concordant individuals (Ct value ≥ 35 for both NP and salivary RT-qPCR) are in upper right white corner . Discordant results (negative for one specimen and positive for the other in RT-qPCR) are on the grey zones of the graphic (NP+/Sal- ; NP-/Sal+).

The sensitivity of the RT-LAMP was calculated by varying the cutoffs of NP or salivary RT-qPCR Ct values (30 to 35) used to determine positive individuals (Fig. 2A). RT-LAMP sensitivity was associated (R^2^ = 0.92) with the salivary RT-qPCR Ct values (sensitivity was 100% when Ct value cutoff < 31 (n = 52)). On the other hand, compared to NP RT-qPCR the sensitivity of the RT-LAMP ranged from 87% (Ct value cutoff < 35) to 89% (Ct value < 30 (R^2^ = 0.61)).

### Performances of RT-LAMP compared to NP and salivary RT-qPCR separately

Salivary RT-LAMP was also compared to RT-qPCR performed on NP and salivary specimens, separately. Firstly, RT-LAMP detected 35 individuals not detected by nasopharyngeal RT-qPCR and missed 4 subjects detected by NP RT-qPCR (Supplementary Table S1). Sensitivity and specificity were 87.1% (95% CI 75.3–98.9) and 91.5% (95% CI 88.8–94.2), respectively (Table 3).

Finally, the RT-LAMP and RT-qPCR methods performed on the same saliva samples were compared (Supplementary Table S2). The sensitivity of RT-LAMP was 91.0% (95% CI 84.2–97.9) and the specificity was 99.5% (95% CI 98.7–100) (Table 3).

### Concordance between saliva and nasopharyngeal specimen using RT-qPCR

After excluding 2 patients with inconclusive NP RT-qPCR results, Gwet’s AC1 showed that both RT-qPCR methods are concordant with an agreement of 87.3% (95% CI, 83.3–91.3%): 371/441 participants were negative and 27/441 were positive for the two specimens (Supplementary Table S3). Discordant results between NP and saliva specimens were observed for 43 participants (10%) (Fig. 2B). Serological analysis performed at least 15 days after RT-qPCRs (n = 5), presence of symptoms compatible with SARS-CoV-2 infection and viral loads strongly suggested that discordant cases were infected cases (Supplementary Table S4). Positive participants identified with each assay (NP RT-qPCR, salivary RT-qPCR and RT-LAMP) are shown in a Venn diagram (Supplementary Figure S1).

### Impact of variants emergence over RT-LAMP detection

Regular in silico alignments of the region targeted by the RT-LAMP test with appearing strain genome sequences permit to identify potential decrease in sensitivity of diagnosis tests.

We tested the potential effect of characteristic mutations of the different SARS-CoV-2 variants of concern (VOC) or variants of interest (VOI) defined by WHO (https://www.who.int/en/activities/tracking-SARS-CoV-2-variants) on the detection of virus by our assay (Table 4). In silico analysis were performed by aligning the six RT-LAMP primers used in the test with all the known variants. In vitro confirmation was also performed for the P1, B1.1.7 and B1.351 variants (manuscript in preparation). No dominant mutation in RT-LAMP primers annealing regions was observed for variants B.1.1.7, B.1.351, C.37, P.1 and most of B.1.617.2 (except for two descendent lineages AY.25 and AY.34). Four variants presenting mutations with concerning prevalence were observed. The mutated VOCs AY.25 (major mutation 26107G > C) and AY.34 (major mutation 26109G > A) and the VOIs B.1.621 and its descendent lineage B.1.621.1 (26158_26161del) presented a prevalence of 6.1%, 0.3% and 0.2%, respectively, of all sequenced specimens over the world and sampled between July to October 2021 (updated on November 10^th^ 2021).

**Table 4:** Variant detection analysis using salivary RT-LAMP assay.

SARS-CoV-2 Variant Name*	Earliest documented samples	WHO definition	Descendent lineages*	RT-LAMP detection	
				In silico analysis**	In vitro analysis***
B.1.1.7/Alpha	United Kingdom, Sep-2020	VOC	Q1–Q8	+	+
B.1.351/Beta	South-Africa, May-2020	VOC	B.1.351.1–B.1.351.5	+	+
B.1.617.2/Delta	India, Oct-2020	VOC	All AY. lineages	+	–
				except for AY.25 and AY.34	
B.1.621/Mu	Colombia, Jan 2021	VOI	B.1.621.1	−	–
C.37/Lambda	Peru, Dec-2020	VOI	C.37.1	+	–
P.1/Gamma	Brazil, Nov-2020	VOC	P.1.1–P.1.17.1	+	+

*Lineages and their descendents were from cov-lineages.org, updated on November 10, 2021. **In silico analysis considered as positive, variants presenting a perfect matched alignment with RT-LAMP primers with a frequency of at least 95% of all sequencing deposed on GISAID during the period of analysis (updated on November 02, 2021). ***RT-LAMP In vitro analysis were performed as described in Method section using referenced commercial samples from Twist Bioscience (B.1.1.7 – ref# 103,926; B.1.351 – ref# 104,043; P.1 – ref# 104,044). ( +) Positive detection; (−) Negative detection; (–) Not determined; VOC, variants of concern; VOI, variants of interest.

## Discussion

SARS-CoV-2 is detected in various human specimens but none of diagnostic test is ideal for COVID-19^19–21^ mass screening. Considering the high specificity of the existing RT-qPCR for SARS-CoV-2 detection, it is relevant to consider an individual as being infected when a RT-qPCR test is positive independently of the specimen^18,22^. Since NP and salivary RT-qPCR technologies were demonstrated to be complementary^14^, we defined a Composite Reference Test (CRTest) to evaluate the diagnostic performances of the RT-LAMP assay.

EasyCOV® is a rapid, extraction-free and connected RT-LAMP salivary test for SARS-CoV-2 detection developed in the beginning of the pandemic burden^23^. The mobile/tablet application EasyCOV Reader® interprets the results of the RT-LAMP assay (positive/negative) and transfers them directly to the sanitary authorities. In addition, this application allows the storage of other data simultaneously (i.e. color parameter of the fluid sample, patient name, date and time of the test, lot number of the assay, serial number of the machine that performed the test, GPS position of the test location). These data can be used in countries or regions to create a map of the geographical distribution of the pandemic. Further, following these data over time enables epidemiologic assessment of the geographical and temporal evolution of the pandemic.

Compared to CRTest in our ambulatory screening context, this RT-LAMP assay showed a good performance (Se = 85.92% and Sp = 99.46%) on the total population as well as on symptomatic and asymptomatic individuals. These performances are of the same order as most of the SARS-CoV-2 diagnostic tests, including NP RT-qPCR^19,20^. By comparison, a recent meta-analysis showed saliva NAAT pooled sensitivity of 83.2% and specificity of 99.2%^14^. The EasyCOV® RT-LAMP test was also recently evaluated against NP RT-PCR, saliva RT-PCR and NP antigenic tests by LeGoff and collaborators^21^. In this study, the authors reported a sensitivity and a specificity of 34% (95%CI 26–44) and 97% (95%CI 96–98), respectively. Their results differed substantially from ours.

The worked performed by LeGoff and collaborators was also carried out in France but a few months later than ours and the prevalence of positive participants were similar between the two studies using NP RT-qPCR test (7% for both studies)^21^. On the other hand, when saliva was used as specimen, the prevalence found by LeGoff was clearly lower compared to our clinical study (9% and 5% against 15.2% and 14.2%) using saliva RT-qPCR and saliva RT-LAMP, respectively. These discrepancies should be explained by either saliva miss processing or difference in protocols for the RT-qPCR and RT-LAMP assays. When compared with our study there are two critical methodologic differences in LeGoff study. The authors used negative and positive controls for each run of RT-qPCRs but they did not perform any kind of control when using RT-LAMP assay. In our work presented here, we systematically used positive and negative controls for each analysis, which validate the correct use or correct storage of the RT-LAMP test.

Besides, LeGoff et al. reported that the result of the RT-LAMP test was performed using a pH sensitive reagent^21^. Actually, the specifications of the EasyCOV® RT-LAMP assay explicitly mention that the use of the intercalant reagent SYBR green is requested to reveal the presence of the amplicons in the reaction tube even for colorimetric readout. LeGoff et al. read by visual observation the coloration of the tube after reaction while we used the dedicated smartphone/tablet application EasyCOV Reader®, which is preconized by the manufacturers to avoid any well-known subjective interpretation of the readout depending on the light of the room and the operator. EasyCOV Reader® was developed to provide objective interpretation of the results of the RT-LAMP test.

SARS-CoV-2 presents a moderate mutation frequency^24–26^. Over the course of evolution, some variants become preponderant in human populations (e.g. “UK variant”-lineageB.1.1.7) and raise widespread concern^27–29^. Genetic variants can lead to false negative SARS-CoV-2 molecular testing if mutations occur in the targeted regions of the primers or probes of the diagnosis tests. We evaluated the frequency of sequenced variants in France and over the World presenting mutations in the annealing region of the RT-LAMP primers during the period of the study of LeGoff and collaborators and ours. No dominant mutation was observed and our analysis shows that 91.9 to 99.1% of all sequenced samples presented 100% of identity with the target sequence of RT-LAMP primers between May 2020 and February 2021 in France and 95.0 and 97.1% over the World (Supplementary Table S6). Further, continuous surveillance allows to evaluate the capability of EasyCOV® to detect the new appearing strains of SARS-CoV-2. Four recent mutated variants (AY.25, AY.34, B.1.621 and B.1.621.1) present mutations in the region targeted by RT-LAMP primers but together they represented only 6.6% of all the sequenced specimens in samples collected in the last four months.

Various other RT-LAMP tests using extraction/purification steps were developed to detect SARS-CoV-2^30–33^. Their sensitivities on NPS samples were equivalent to that of RT-qPCR^30–32^. Nagura-Ikeda et al.^34^ compared six different NAATs (including one RT-LAMP) and an antigen test using self-collected saliva from hospitalized patients. The sensitivities of the evaluated tests for SARS-CoV-2 detection were 50.5 to 81.6% (70.9% by RT-LAMP) using NAATs and 11.7% using the antigen test. Yokota et al.^8^ conducted a mass-screening study on asymptomatic individuals. Saliva was analyzed by both RT-qPCR and RT-LAMP. Excepted four samples tested negative by RT-LAMP and positive by RT-qPCR (Ct values ranging from 36.0 to 37.3), the two techniques displayed good concordance.

Further, a recent meta-analysis found a high pooled sensitivity for antigen rapid diagnostic tests (Ag-RDTs) when using NP samples and a Ct cutoff for RT-qPCR < 30 (79.90% (95%CI 70–87). However, for saliva specimen the pooled sensitivity found was 37.9% (95%CI 12–74) independently of the Ct cutoff used for the reference standard RT-qPCR^35^. Despite the rapidness and the low-cost of Ag-RDTs, the observed performances in the meta-analysis were lower compared to EasyCOV® and NP sampling is still required for Ag-RDTs to achieve even moderate diagnostic performances.

We also compared the salivary RT-LAMP to the salivary RT-qPCRs separately. The RT-LAMP test presents good performances compared to salivary RT-qPCR and its sensitivity was associated with the Ct value cutoff in salivary RT-qPCR. The six misdiagnosed subjects by RT-LAMP presented Ct values ≥ 31, reflecting the higher LOD (limit of detection) of the RT-LAMP compared to the RT-qPCR. Our results corroborated previous results comparing salivary RT-LAMP and RT-qPCR performances and demonstrated similar performances between the two methods^36–39^.

Compared to nasopharyngeal RT-qPCR only, the RT-LAMP showed a weaker specificity and PPV, reflecting the negative individuals misclassified by NP RT-qPCR but also detected positive by salivary RT-qPCR. Further, viral load in NPS samples was weakly associated to RT-LAMP sensitivity as there was no correlation between salivary and NP RT-qPCR Ct values (Spearman correlation = 0.19), suggesting different saliva and NPS viral loads, corroborating previous works^6,40^.

Further, we analyzed 443 saliva and NP paired samples using optimized RT-qPCR protocols to deal with the two different specimens. A total of 71 individuals were positive from which 43 had discordant results between NP and salivary RT-qPCR. Most (n = 39) were detected positive only in saliva while clearly negative in NP RT-qPCR without Ct value. Five individuals proceeded to a serological test and all were positive, corroborating the salivary RT-qPCR results and suggesting an infection at sampling. Contrarily, four participants were positive in NP RT-qPCR only. For three of them, the presence of the viral RNA at low loads in saliva was observed (Ct value ≥ 35). Different kinetics of virus replication in saliva and NP have already been described^6,7^. In our study, NP RT-qPCR broadly underestimated the number of positive cases detected using salivary RT-qPCR and salivary RT-LAMP. Low sensitivities have also been reported earlier^19,20^ and might reflect the difficulty of the NPS sampling. Collecting saliva is a non-invasive and easy procedure in contrast with NP or OP swabs. In ambulatory practice, the use of saliva to detect SARS-CoV-2 has several advantages. It avoids individual’s discomfort, minimizes healthcare personnel exposure and well accepted by population. Accordingly, we showed the feasibility of saliva self-sampling in asymptomatic or mildly symptomatic outpatients. Despite NPS being the most used sampling method, saliva was demonstrated to be a reliable specimen for the detection of SARS-CoV-2 in other several studies^6,8,9,11,21,32,41–43^ and our results showed that saliva presents a superior performance for COVID-19 diagnosis using RT-qPCR compared to NPS.

The rapidness, easiness and performances of the RT-LAMP EasyCOV®, combined with acceptability of saliva sampling^2,3^, confirm the usability (no need of laboratory facilities) of extraction-free salivary RT-LAMP tests for SARS-CoV-2 screening in a point of care setting. The secured internet connectivity of this device allows the real-time management of crowds in the context of social events, border control and professional and educational plants in which a negative test would be required for accessing controlled sites. This assay is already employed in airports and country borders as well in companies to test employers. Altogether, our results demonstrate that the rapid salivary RT-LAMP is an operational alternative for SARS-CoV-2 detection in the context of safe economic and social reopening.

## Materials and methods

### Study design and population

In this monocentric diagnostic study, adults coming to the COVID-19 screening centre of University Montpellier Hospital between 22th May and 7th of October 2020 with COVID-19 compatible symptoms and/or being contact-cases of confirmed COVID-19 cases were prospectively invited to participate.

Symptoms, risk factors, medical history and treatments were informed on a self-administered questionnaire under supervision of a practitioner. A participant was considered asymptomatic if no symptom was declared at the time of sampling.

Participants were requested to self-collect 2 mL of saliva by salivating in a 50 mL polystyrene tube and then proceeded to a simultaneous and mandatory NPS sampling by a trained nurse. Saliva samples were immediately stored at 4 °C and sent within 3 h to the research laboratory Sys2Diag. Once at Sys2Diag, each sample was separated into two new tubes and routed to two different laboratory rooms for RT-qPCR and RT-LAMP analysis, following the international laboratory-guidance, by independent biologists blinded of the infectious status of the participants and the results of NP RT-qPCR. NPS were discharged in 1 mL viral transport medium (VTM) and samples were tested by RT-qPCR by independent blinded virologists at the hospital.

The study and all subsequent amendments was approved by a French ethic committee (CPP-Ile de France XI) on April 03, 2020. The study was registered at www.clinicaltrials.gov (NCT04337424). All methods were performed in accordance with relevant guidelines and regulations. All participants signed informed consent prior to participating.

### RNA extraction from nasopharyngeal sampling and RT-qPCR SARS-CoV-2 virus detection

Prior to RNA extraction, 200 µL of VTM supplemented samples were inactivated by mixing with 200 µL of ATL Lysis buffer (Qiagen). RNA extractions were performed on 200 µL of inactivated samples and experiments were carried out using three different processes, according to their availability at University Hospital: (1) Extraction on the Alinity m system (Abbott) using the Alinity m Sample Prep Kit 2, Alinity m Lysis Solution, and Alinity m Diluent Solution (Abbott) followed by RT-qPCR on the Alinity m system using the proprietary Alinity m SARS-CoV-2 AMP Kit targeting RdRp and N genes; (2) Automatic extraction on a Starlet platform (Hamilton) using the Starmag 96 Universal kit (Seegene) followed by RT-qPCR targeting the RdRp, E and N viral genes on a CFX 96 (Biorad) using the Allplex 2019-nCov assay kit (Seegene); (3) Extraction on MGISP-NE32 (MGI) was carried out using the MGIEasy Nucleic Acid Extraction Kit (MGI) followed by RT-qPCR on a LightCycler480 (Roche) using either Real time fluorescent RT-PCR (RdRp probe) (BGI) or the RT-PCR Argene SARS-CoV2 R-gene kit (N and RdRp Probes) (BioMérieux).

### RNA extraction from saliva and RT-qPCR SARS-CoV-2 virus detection

RNA extraction from saliva was performed as described earlier^23^. Briefly, samples were treated in the presence of DTT (10 mM) for 30 min at room temperature. Then, RNA extraction was performed using Nucleospin Dx Virus Kit (Macherey Nagel). 2 μL of purified RNA were added to Invitrogen superscript III Platinium One step reaction mix (#11,732,020) containing 1X reaction mix, MgSO4 (0.8 mM) a DNA‐primer mix aiming two RdRp targets (IP2 and IP4). Primers and probes (nCoV_IP2 and nCoV_IP4) were designed to target the RdRp gene spanning nt 12,621–12,727 and 14,010–14,116 (positions according SARS-CoV, NC_004718)^44^. Real-time detection and thermal cycling conditions were identical to previously described^23^. Saliva samples were analyzed in triplicate and RT-qPCR Ct values were the mean of these results. An absence of amplification on replicates was expressed as Ct = 40 which is the maximum value obtained by our method.

### Index test: Saliva pretreatment and direct RT-LAMP for SARS-CoV-2 virus detection

Rapid direct detection of SARS-CoV-2 by RT-LAMP was performed using EasyCOV® (SkillCell) following manufacturer’s instructions. Briefly, the 40 min test consists in three simple steps: firstly, 200 µL of saliva are added to tube one and incubated for 10 min at 80 °C for pretreatment. Then 3 µL from tube one are transferred to tube two containing the RT-LAMP reaction mix for viral RNA amplification. Tube two is incubated for 30 min at 65 °C. A colorimetric instantaneous result is obtained after addition of 1 µL of revelation reagent to tube two. Positive samples for SARS-CoV-2 turn yellow while negative ones remain orange. The final color is read by naked eye or automatically interpreted by EasyCOV® Reader application (VOGO, France) which displayed and recorded the result in a health secured online database.

For each experiment, we performed positive and negative controls using a SARS-CoV-2 synthetic RNA (CODEX DNA, SC2-RNAC0500), well-characterized positive and negative saliva samples and no template control (H_2_O).

The robustness of the salivary RT-LAMP test was assessed. Replicability of experiment was performed testing each clinical sample in triplicate. The sensitivity was also calculated with a random sampling without replacement on the triplicated tests using the bootstrap method (5000 sample tests) (Table S5). Performances obtained using bootstrap (Supplementary Table S5) or single point analyses (data not shown) were similar to those considering at least two out of three replicates as positive. For the reproducibility of the RT-LAMP test, 30 subjects were randomly asked to be sampled twice in two separate tubes at the same moment. Each sample was analysed separately and the raw concordance between the two samplings was of 96.9% (data not shown).

### EasyCOV connected test via Reader© Apps

EasyCOV Reader® is a mobile device application (iOS/Android) which assists the interpretation of EasyCOV® result. At the end of the assay, the colored tube two is photographed by an operator using the application. The software automatically determines the position of the fluid sample in the tube and the position of the tube on the reference template. From a colorimetric analysis based on the HSV space (Hue, Saturation Value) of the fluidic sample (patent pending) and taking into account the brightness parameters, the software provides an instant result (virus detected/virus not detected). EasyCOV Reader® records patient’s information and registers the image of tube two. The result is real time displayed and recorded in a secured online database. Then any authorized entity can access a real time desktop to monitor the results of ambulatory testing.

### SARS-CoV-2 variant analysis

The sequences of SARS-CoV-2 variant genomes were downloaded with their metadata from the data base GISAID^45^ on November 02, 2021. Then genomes were aligned with the EasyCOV primers with the software MUMmer4 (version 4.0.0 release candidate 1). For each pair of primer/genome the larger alignment was kept. The alignment data and Metadata were merged with Bash (version 5.0.3) and python (version 3.7.3) scripts. Finally, with an R script (version 4.0.5) it was possible to isolate genomes of interest; those from variants found in *Homo sapiens*, with high coverage (less than 1% of Ns) and complete (with more than 29 kb). It was also possible to identify for each month, genomes of interest aligning without any mismatch or insertion or deletion with the full-length primer sequences in the expected positions.

### Sample size

The sensitivity of NP RT-qPCR for SARS-CoV-2 detection was estimated at 71%^19,20^. Considering a sensitivity for the RT-LAMP of 70%, with an accuracy of ± 10% of the 95% confidence interval (CI), we calculated that 80 participants positive for SARS-CoV-2 infection had to be included. With an estimated prevalence rate of 15% at the screening center of our Hospital University, we thus aimed at recruiting about 533 individuals.

### Statistical analysis

Values were expressed as mean + /− standard deviation (SD) for continuous variables and number with percentages for categorical ones. Comparisons of the clinical characteristics between positive and negative patients were performed with Student or non parametric (Wilcoxon-Mann–Whitney, WMW) tests for continuous variables and with chi-2 test for categorical variables.

The performance of the diagnostic tests was evaluated by their sensitivity (Se) and specificity (Sp) with their 95% confidence interval. Sensitivity was the proportion of positive index test in the infected population and specificity the proportion of negative in the non-infected population, according to the reference diagnosis. Since our study was carried out in a real screening context, the positive and negative predictive values were also calculated with their 95% CI for the index test. Accuracy (true positive plus true negative cases divided by the total number of participants) was also reported. Sub-group analysis was performed according to the presence/absence of symptoms.

The concordance between nasopharyngeal and salivary RT-qPCR was assessed by calculating the Gwet’s agreement coefficient (AC1) as positive and negative distributions were unbalanced. All statistics were performed using SAS Enterprise Guide, v7.3 (SAS Institute Inc, Cary, NC, USA).

## Supplementary Information

Supplementary Information.
