# Supplementary material for: Performances of rapid and connected salivary RT-LAMP diagnostic test for SARS-CoV-2 infection in ambulatory screening
Source: Sci Rep. 2022 Feb 18;12:2843. doi: 10.1038/s41598-022-04826-7 (PMC8857239; doi:10.1038/s41598-022-04826-7)
Supplement: Supplementary file 1 — Supplementary Information. [file 41598_2022_4826_MOESM1_ESM.pdf]

Performances of rapid and connected salivary RT-LAMP diagnostic test for SARS-CoV-2 infection in ambulatory screening

Francisco Santos Schneider[1,2¶], Laurence Molina[1¶], Marie-Christine Picot[3,4¶], Nicolas L'Helgoualch[1], Julien Espeut[1,2], Pierre Champigneux[1], Mellis Alali[1], Julie Baptiste[1,2], Lise Cardeur[1], Christophe Carniel[5], Martin Davy[1,2], Daniel Dedisse[5], Benjamin Dubuc[1], Hugo Fenech[1], Vincent Foulongne[6], Carole Fruchart Gaillard[7], Florence Galtier[4], Alain Makinson[8,9], Grégory Marin[3], Raissa Medina Santos[1,10], David Morquin[8,9], Alimata Ouedraogo[1], Alexandra Prieux Lejeune[1,2], Marine Quenot[1], Pierre Keiflin[5], Francisco Checa Robles[1], Carolina Rodrigues Rego[1,10], Nicolas Salvetat[1], Charline Trento[1], Diana Vetter[1], Franck Molina[1*&] and Jacques Reynes[8,9&]

Supplementary Information

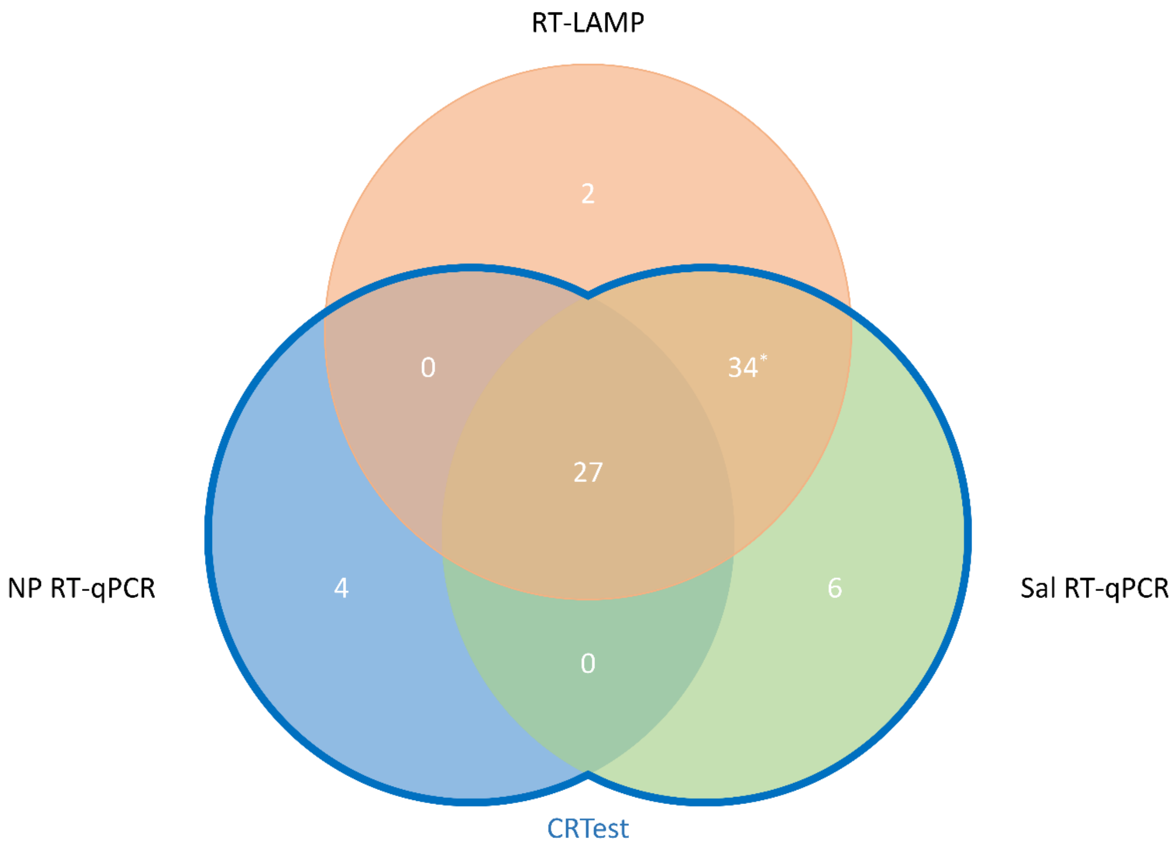

Figure S1. Venn diagram of the cross evaluation of three methods to address infectiousness of an individual. RT-qPCR, reverse transcription quantitative polymerase chain reaction; RT-LAMP, reverse transcription loop-mediated isothermal amplification; NP, nasopharyngeal; Sal, salivary; CRTest, composite reference test. The Venn diagram describes only the positive participants found using in each assay: NP RT-qPCR, salivary RT-qPCR and RT-LAMP. The blue line showed the positive results corresponding to the Composite Reference Test (CRTest). *One individual had inconclusive result on NP RT-qPCR and was positive for salivary RT-qPCR and RT-LAMP.

Supplementary Table

		Nasopharyngeal RT-qPCR		
		Negative or C_t value ≥ 35	Positive C_t value < 35	Total
Salivary RT-LAMP	Negative	375	4	379
	Positive	35	27	62
	Total	410	31	441†

Supplementary Table S1. Concordance table of salivary RT-LAMP results according to nasopharyngeal RT-qPCR results. Data are reported as number of individuals. RT-qPCR, reverse transcription quantitative polymerase chain reaction; RT-LAMP, reverse transcription loop-mediated isothermal amplification; C_t, cycle threshold. † 2 of 443 participants had inconclusive results by nasopharyngeal RT-qPCR (one was positive with salivary RT-qPCR and RT-LAMP and the other was negative with both methods).

		Salivary RT-qPCR		
		Negative or C_t value ≥35	Positive C_t value <35	Total
Salivary RT-LAMP	Negative	374	6	380
	Positive	2	61	63
	Total	376	67	443

Supplementary Table S2. Concordance table of RT-LAMP results according to results of RT-qPCR on saliva samples. Data are reported as number of individuals. RT-qPCR, reverse transcription quantitative polymerase chain reaction; RT-LAMP, reverse transcription loop-mediated isothermal amplification; Ct, cycle threshold.

		Nasopharyngeal RT-qPCR		
		Negative or Ct value ≥35	Positive (Ct value <35)	Total
Salivary RT-qPCR	Negative or Ct value ≥35	371	4	375
	Positive (Ct value <35)	39	27	66
	Total	410	31	441†

Supplementary Table S3. Concordance between nasopharyngeal RT-qPCR and salivary RT-qPCR. Data are presented as number of individuals. RT-qPCR, reverse transcription quantitative polymerase chain reaction; Ct, cycle threshold. † 2 of 443 participants had inconclusive results by nasopharyngeal RT-qPCR.

Subject ID	C_t Value		Sal RT-LAMP	Clinical symptoms						Serology
	Sal RT-qPCR	NP RT-qPCR		Fever	Myalgia	Headache	Vertigo	Ageusia	Anosmia	
Individuals presenting a positive NP RT-qPCR and negative salivary RT-qPCR (n=4) †										
147	35.4	23.1	-	+	+	-	-	+	+	..
341	35.1	31.3	-	-	-	-	-	-	-	..
459	ND	33.2	-	-	+	+	+	+	+	..
594	34.6	27.2	-	-	-	-	-	-	-	..
Individuals presenting a positive salivary RT-qPCR and negative NP RT-qPCR (n=39) ‡										
170	30.8	ND	+	-	-	+	-	-	-	..
171	22.8	35.6	+	-	-	-	-	+	+	..
181	30.4	ND	+	+	-	-	-	-	-	..
191	28.9	ND	+	+	+	+	-	-	+	..
193	29.4	ND	+	-	-	-	-	-	-	..
201	25.5	ND	+	-	-	-	-	-	-	..
210	20.5	ND	+	-	-	+	-	-	-	+
211	33.2	ND	+	-	-	-	-	-	-	..
214	27.1	ND	+	-	-	-	+	+	-	..
215	29.4	ND	+	-	+	-	-	-	-	..
235	27.3	ND	+	-	-	+	-	-	+	+
261	22.1	ND	+	+	-	+	+	-	-	..
264	31.4	36.4	+	-	+	+	-	-	+	..
275	31.7	ND	-	-	-	-	-	-	-	..
279	33.0	ND	-	-	+	-	+	+	+	+
284	34.0	ND	-	+	+	+	-	-	-	..
285	29.4	ND	+	-	-	-	-	-	-	+
287	19.0	ND	+	+	+	+	-	-	-	..
308	28.9	ND	+	-	+	-	-	-	-	+
328	31.8	ND	+	+	-	+	-	-	-	..
358	21.2	ND	+	-	-	-	-	-	-	..
396	30.0	ND	+	-	-	+	-	+	+	..
408	33.2	ND	+	-	-	+	-	+	+	..
412	30.4	35.5	+	-	-	+	-	-	-	..
419	28.3	34.7	+	-	-	-	-	-	-	..
425	30.3	ND	+	-	-	-	-	-	-	..
426	31.6	ND	+	-	-	-	-	-	-	..
433	23.1	36.9	+	-	-	-	-	-	-	..
464	29.1	36.5	+	+	+	+	-	+	+	..
509	31.5	ND	-	-	-	-	-	-	-	..
535	26.0	ND	+	-	+	-	-	+	+	..
539	31.3	ND	-	-	-	-	-	-	-	..

554	30.4	ND	+	-	-	-	-	-	-	..
556	26.2	36.1	+	-	-	-	-	+	+	..
570	26.1	ND	+	-	-	-	-	-	-	..
589	31.8	ND	+	+	+	+	+	-	-	..
591	16.0	ND	+	-	-	-	-	-	-	..
597	26.1	36.0	+	-	+	+	+	+	+	..
622	32.5	ND	-	-	-	-	-	+	+	..

Supplementary Table S4. Description of viral loads, clinical symptoms and serology results in individuals with discordant results between salivary and NP RT-qPCT (n=43). Ct, cycle threshold; Sal; salivary; NP, nasopharyngeal; RT-qPCR, reverse transcription quantitative polymerase chain reaction; RT-LAMP, reverse transcription loop-mediated isothermal amplification; ND: no detected amplification signal; (+) Positive salivary RT-LAMP or Presence of symptom; (-) Negative salivary RT-LAMP or Absence of symptom; (..), Data not collected/available. † Among the 4 individuals with negative salivary RT-qPCR and positive NP RT-qPCR, three individuals had a salivary Ct value of 35 (threshold value). Two presented symptoms of COVID-19 while two were asymptomatic. None performed the serological test. ‡ Among the 39 individuals with negative NP RT-qPCR and positive salivary RT-qPCR, eight samples had NP Ct values ≥35 and 31 had no detectable amplification signal in NP RT-qPCR. Among these 31 individuals, a broad range of viral load was observed in saliva (Ct values between 16.0 and 34.0). Out of the 39 discordant individuals, 29 were symptomatic. Four of them performed serological analysis and all were positive. Among the 10 asymptomatic individuals, one performed serological analysis and was positive.

Salivary RT-LAMP test	N	Mean (± SD)	Median (Min;Max)	[Q25;Q75]
Sensitivity	5000	83.55 (± 1.96)	83.57 (76.30;91.30)	[82.22;84.89]
Specificity	5000	98.66 (± 0.26)	98.67 (97.80;99.72)	[98.46;98.86]

Supplementary Table S5. Performances of a random sampling without replacement on the triplicated tests using the bootstrap method (5000 sample tests). Under operating conditions, the EasyCOV® test will only be performed once (no in triplicate). In order to assess the robustness of the results by taking only one measurement out of the 3, performances were also calculated by randomizing one RT-LAMP result per patient out of the three (triplicate) : 5000 bootstrapped samples were thus created, with replacement and sensitivities and specificities were calculated for each sample.

Period of analysis (month)	World			France		
	Ngi	Npm	Ppm (%)	Ngi	Npm	Ppm (%)
May-20	19046	18330	96.24	177	172	97.18
June-20	21706	21096	97.19	62	57	91.94
July-20	29336	28240	96.26	113	112	99.12
August-20	29538	28345	95.96	334	330	98.80
Sept.-20	33837	32709	96.67	1094	1078	98.54
Oct.-20	53196	51208	96.26	790	772	97.72
Nov.-20	72816	70087	96.25	865	822	95.03
Dec.-20	101822	97024	95.29	713	671	94.11
Jan.-21	177915	169099	95.04	3384	3276	96.81
Feb.-21	196591	187923	95.59	3364	3201	95.15
March-21	310886	296870	95.49	9501	9014	94.87
April-21	310811	293959	94.58	6282	5934	94.46
May -21	246664	232214	94.14	4189	3937	93.98
June-21	195673	183276	93.66	3093	2947	95.28
July-21	362617	331017	91.29	13153	12497	95.01
August-21	564449	501765	88.89	12208	11338	92.87
Sept.-21	429403	375255	87.39	5643	5194	92.04
Oct.-21	151371	136851	90.41	1876	1776	94.67

Supplementary Table S6. Frequency of sequenced variants in France and over the World presenting mutations in the annealing region of the RT-LAMP primers since the appearance of the SARS-CoV-2. Ngi, number of sequenced genomes of interest; Npm, number sequences presenting a perfect match alignment; Ppm, percentage of perfect matching (npm/ngi*100).
